# In vivo detection of pathologic α- synuclein and TDP-43 in the skin and olfactory mucosa of a patient with Parkinson’s disease-like phenotype carrying the p.Thr272Serfs*10 GRN mutation

**DOI:** 10.1186/s13024-026-01000-9

**Published:** 2026-10-01

**Authors:** Paola Caroppo, Vittoria Aprea, Cristina Villa, Ilaria Linda Dellarole, Aurora Romeo, Giacomina Rossi, Arianna Ciullini, Anna Burato, Sara Prioni, Marina Grisoli, Giuseppe Legname, Giulia Simmini, Alessia Luppino, Samanta Mazzetti, Fabio Moda, Grazia Devigili

**Affiliations:** 1 Neurology 8 – Dementia and Degenerative Diseases of CNS Unit, Fondazione IRCCS Istituto Neurologico Carlo Besta, Milan, Italy; 2 SSD Laboratory Medicine-Laboratory of Clinical Pathology, Fondazione IRCCS Istituto Neurologico Carlo Besta, Milan, Italy; 3 Laboratory of Prion Biology, Department of Neuroscience, Scuola Internazionale Superiore Di Studi Avanzati (SISSA), Trieste, Italy; 4 Clinical Neuropsychology Unit, Fondazione IRCCS Istituto Neurologico Carlo Besta, Milan, Italy; 5 Neuroradiology Unit – Fondazione IRCCS Istituto Neurologico Carlo Besta, Milan, Italy; 6 Neurology 1 Unit, Fondazione IRCCS Istituto Neurologico Carlo Besta, Milan, Italy; 7 Department of Medical Biotechnology and Translational Medicine, University of Milan, Milan, Italy

**Keywords:** FTD, Frontotemporal dementia, *GRN*, Parkinson’s disease, TDP-43, α -synuclein, Skin biopsy, SAA

## Abstract

We describe a patient carrying the *GRN* p.Thr272Serfs*10 mutation, who presented with parkinsonism at onset and later developed prodromal frontotemporal dementia, in whom seed amplification assay on olfactory mucosa and skin analyses suggest mixed TDP-43 and α-synuclein co-pathology, highlighting how combining these techniques could provide a comprehensive approach to investigate the underlying neuropathological process.

**Supplementary Information:**

The online version contains supplementary material available at https://doi.org/10.1186/s13024-026-01000-9.

**To the Editor** **,**

Co-pathology is an important topic of research in neurodegenerative disorders linked to misfolded proteins accumulation. TAR DNA-binding protein 43 (TDP-43) proteinopathy is the pathological signature of frontotemporal dementia (FTD) linked to *GRN* mutations, representing almost the 35% of familial FTD [1]. Seed amplification assays (SAAs) have shown high sensitivity in detecting misfolded TDP-43 (TDP-43_SAA) in the olfactory mucosa (OM) of genetic FTD [2]. Similarly, SAA was employed to detect misfolded α-synuclein SAA (αSyn_SAA) in OM of patients with synucleinopathies, such as Parkinson’s disease (PD) [3]. In this report we describe a patient with PD-like phenotype and prodromal FTD carrying the *GRN* p.Thr272Serfs*10 mutation, in whom we hypothesize mixed α-synuclein/TDP-43 co-pathology, based on OM and skin analysis, suggesting a possible diagnosis of *GRN*-related parkinsonism with α-synuclein co-pathology. The patient presented at the age of 59 with a right-lateralized bradykinetic-rigid syndrome. Positive SPECT-DaT SCAN (Fig. 1c) and a 45% improvement at MDS-UPDRS Part III score with L-Dopa confirmed the initial diagnosis of PD. He later developed mild depression, RBD-like sleep disturbances, orthostatic hypotension, frontal executive and mild behavioural alterations and impaired social cognition (Supplementary Table 1) consistent with prodromal FTD, on the basis of recent recommendations [4]. MRI showed mild diffuse cortical atrophy (Fig. 1a, b) and FDG-PET (Fig. 1d) revealed increased basal ganglia uptake without cortical hypometabolism. MIBG scintigraphy confirmed cardiac sympathetic denervation (Fig. 1e). Patient’s family history was relevant for FTD developed by his mother who carried the p.Thr272Serfs*10 mutation in the *GRN* gene; the patient underwent genetic testing which revealed the same mutation. OM collection and skin biopsy were performed after 3 years from the onset of the bradykinetic-rigid syndrome, following written consent for all research analysis.

OM sample collection, preparation and extensive methods of SAA and skin analyses are reported in the Supplementary materials.

αSyn_SAA was performed in 384-well plates with 49 µL reaction mix (0.1 mg/mL rec-αSyn, 40 mM HEPES pH 8.0, 170 mM sodium citrate, 20 µM ThT), two silica beads, and 1 µL OM sample. Plates were shaken (1 min, 600 rpm) and incubated (1 min, 42 °C) cyclically in a FLUOstar OMEGA reader; ThT fluorescence (Ex 450 ± 10 nm, Em 480 ± 10 nm) was recorded every 30 min.

TDP-43_SAA was performed with modified protocol of Fontana et al., 2024 [2], in 96-well plates with 96 µL reaction mix (0.05 mg/mL truncated TDP-43, 40 mM Tris-HCl pH 8, 500 mM Guanidine-HCl, 0.002% SDS, 10 µM ThT), 4 µL OM sample, and one 3 mm glass bead. Plates were sealed and incubated at 42 °C in a FLUOstar CLARIOstar reader with alternating shaking (15 s, 100 rpm) and incubation (30 min). ThT fluorescence was measured every 30 min.

Skin biopsies have been performed in 3 body sites (cervical, thigh and distal leg) and processed as previously described [5]. Double immunofluorescence was performed with different primary antibodies mixes: anti-p-syn or p-S409/410 TDP-43 and anti-PGP 9.5 or anti-CD68 (Supplementary materials). Positive controls were performed on human brain sections (Supplementary Fig. 1a) for p-TDP-43 and negative control in healthy subjects for p-TDP-43 (Supplementary Fig. 1b, c) and p-syn (Supplementary Fig. 1d, e).

SAA on OM detected both TDP-43 and αSyn pathological seeds, suggesting the concurrent presence of both protein aggregates (Fig. 1, f). In skin samples, p-syn deposits were extensively analysed for their relationship with PGP9.5-positive nerve fibers (Fig. 1, g-j’), with similar aspect in all three body sites analysed. Despite few and small aggregates were present inside nerve fibers surrounding sweat gland secretory coils (Fig. 1, g), arterioles (Fig. 1, h) and *arrector pilorum* muscle (Fig. 1, k), some cells stained for p-syn were observed in close proximity to nerve fibers (Fig. 1, h, h’) and in dermis (Fig. 1, j, j’). To characterize these cells, we performed double staining for p-syn and vimentin as a marker of fibroblast and CD68 for macrophages and dendritic cells. First, we observed a consistent amount of arborized dendritic cells in the epidermal layer (Fig. 1, i-i’’), supporting an inflammatory epidermal state. Furthermore, we found colocalization of p-syn and CD68 in arborized cells in close proximity with sweat gland duct (Fig. 1, l-l”).

For p-TDP-43 we observed a subset of positive dermal cells, that were also in close contact with the PGP9.5 positive-fibers surrounding a sweat gland (Fig. 1 m, m’) and with nerve bundles (Fig. 1 n, n’). As previously described for amyotrophic lateral sclerosis [6], p-TDP-43 was revealed in vimentin-positive fibroblast (Fig. 1 o-o’’), but also in some macrophages stained for CD68 (Fig. 1 p, q).

To our knowledge, only one study reported SAA detection of synucleinopathy in the CSF of a FTD-TDP case linked to a *TBK1* mutation, without clinical parkinsonism, supporting the use of this technique to investigate co-pathology [7]. Remarkably, we demonstrated that TDP-43_SAA assay could identify TDP-43 pathology in prodromal stages of FTD disease as well as in presymptomatic carriers, as recently described [8] strongly supporting its use in larger cohorts of presymptomatic/prodromal cases.

Findings from skin samples were consistent with SAA results and, more intriguingly, revealed the presence of both p-TDP-43 and p-syn deposits within dermal cells, suggesting a peculiar neuropathological pattern.

To date, only one study has reported the presence of p-syn deposits in the skin of a PD presymptomatic *GRN* carrier [9]. These findings are similar to those observed in the present study, however, by applying confocal microscopy, we were able to further characterize the localization of p-syn deposits within nerve fibers, fibroblasts and CD68-positive cells, and for the first time, to assess p-TDP-43 immunoreactivity in skin biopsies. This study provides the first evidence of p-TDP-43 immunoreactivity detection in the skin of a patient carrying *GRN* mutation. Interestingly, p-TDP-43 immunoreactivity was also detected within macrophages in areas of enhanced inflammation, including arborized CD68 positive dermal macrophages and dendritic cells in the epidermis. Rather than indicating intrinsic expression or aggregation, this signal may reflect processes such as protein uptake, phagocytosis, or an inflammatory response. These observations raise the possibility that GRN dysfunction, by affecting inflammatory regulation, may be associated with cellular stress states favouring protein misfolding, including TDP-43 and α-synuclein, as supported by recent experimental findings [10] potentially extending to peripheral tissues such as the skin. Certainly, this hypothesis of a biological link between *GRN*-associated inflammatory processes and protein aggregation in peripheral tissues warrants further investigations. Our findings, on a single case report, of course should be interpreted with caution. Nevertheless, the combined use of SAA assays and skin analysis could represent an integrated approach for investigating the underlying nature of the neuropathological process also at early stages of disease, enhancing clinical understanding with potential implications for future therapeutic approaches.

**Fig. 1:**
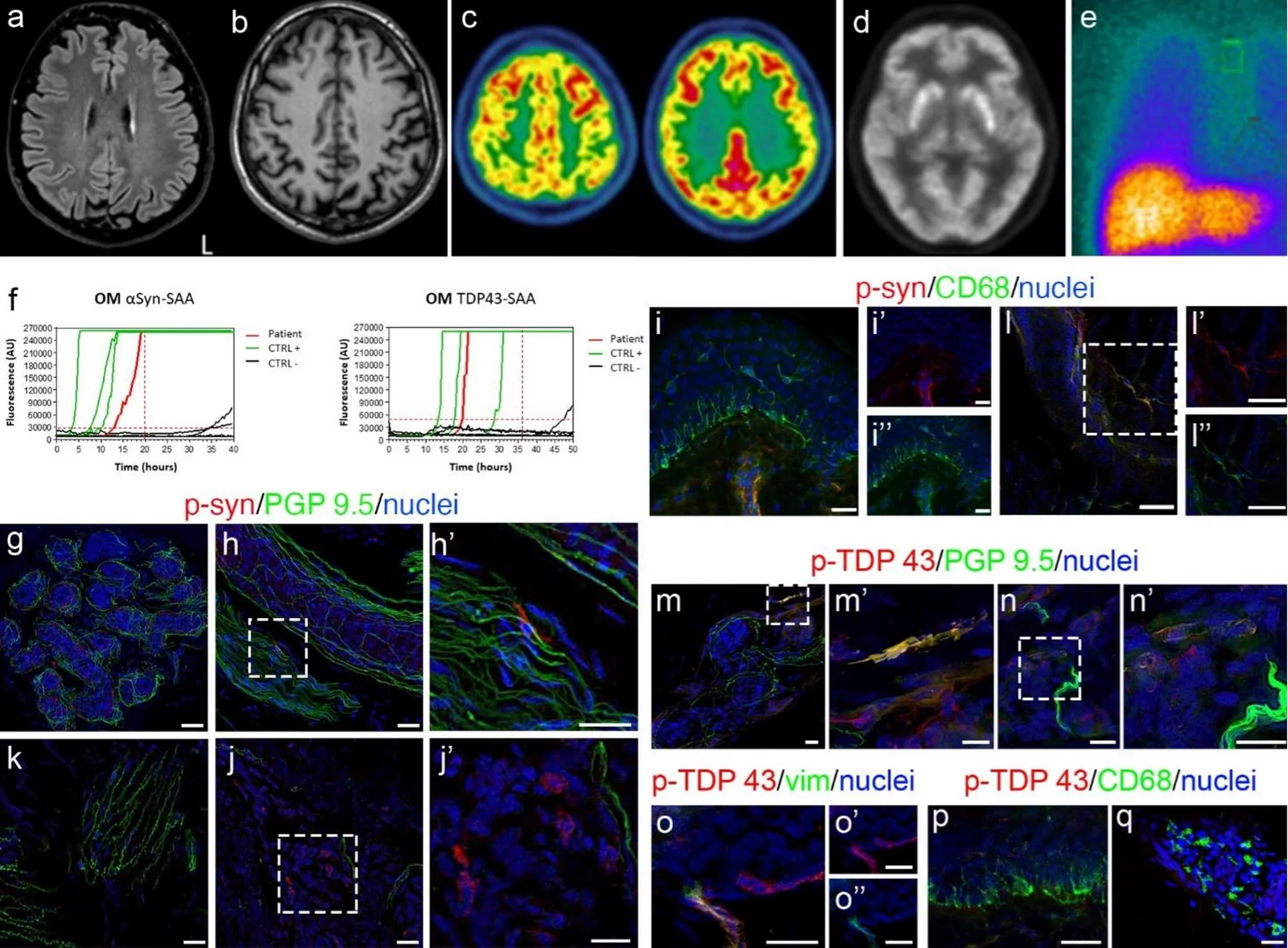
Neuroradiological features (**a-e**), OM_SAA (**f**) and skin analysis (**g-q**).** a**,** b**: MRI showing mild frontal and parietal atrophy with left predominance; **c**,** d**: FDG-PET showing normal cortical metabolism and increased uptake in the basal ganglia; **e**: Cardiac-MIBG showing sympathetic denervation; **f**: α-synuclein (left) and TDP-43 (right) seeding activities detected in the OM, CTRL - = negative controls (OM samples from four healthy subjects), CTRL + = positive controls (OM samples from three patients with PD for αSyn_SAA and from one patient with FTD and two patients with spinal-onset ALS for TDP43_SAA); **g-l’’**: Confocal analysis of p-syn deposits in skin biopsy. **g-j’:** double staining with PGP9.5 shows few pointed deposits in the three autonomic structures, sweat gland (g), arteriole (h) and *arrector pilorum* muscle (k). Furthermore, some p-syn-positive cells are visible in close proximity with nerve fibers (h, h’) and in dermis (j, j’). **i**,** l’’**: CD68 staining shows an consistent amount of arborized dendritic cells in the epidermis (i-i’’) and some p-syn and CD68 arborized cells in close proximity with sweat gland duct (l-l’’); **m-q**: Confocal analysis of p-TDP-43 deposits in skin biopsy; **m- n’**: p-TDP-43-positive cells are visible in the dermis (m, m’) and also in close contact with nerve fibers (n, n’); **o-o”**: vimentin-positive fibroblast shows the presence of a punctate p-TDP-43 staining in the cytoplasm; **p**,** q**: p-TDP-43 is present also in the dendritic cells of the epidermis (p) and in the macrophages CD68-stained (q). Scale bar g, h, k-l”, m, n 30 μm; h’, j’, m’, n’, p, q 20 μm. Vim: vimentin

## Supplementary Information

Below is the link to the electronic supplementary material.

Supplementary Material 1

Supplementary Material 2

## Data Availability

All data generated or analysed during this study are included in this published article.
