# Supplementary material for: In vivo detection of pathologic α- synuclein and TDP-43 in the skin and olfactory mucosa of a patient with Parkinson’s disease-like phenotype carrying the p.Thr272Serfs*10 GRN mutation
Source: Mol Neurodegener. 2026 Oct 1;21:61. doi: 10.1186/s13024-026-01000-9 (PMC13632330; doi:10.1186/s13024-026-01000-9)
Supplement: Supplementary file 1 [file 13024_2026_1000_MOESM1_ESM.docx]

**SUPPLEMENTARY MATERIALS**

***In vivo* detection of pathologic α- synuclein and TDP-43 in the skin and olfactory mucosa**

**of a patient with Parkinson’s disease-like phenotype carrying the p.Thr272Serfs*10 GRN mutation.**

**Methods:**

**OM sample collection and preparation for SAA analyses**

The OM sample of the patient was obtained by a specialized otolaryngologist using a fiberoptic rhinoscope to locate the OM. The medial septal wall, just above the middle turbinate of both nostrils, was gently brushed with a special flocked swab (FLQBrush^TM^ Copan Italia, Brescia, Italy), as previously described [https://doi.org/10.1186/s40035-019-0164-x. https://doi.org/10.1186/s13024-021-00491-y]. After collection, the swab was immersed in 9 mL of saline solution, vortexed and then centrifuged at 800×g for 20 min at 4 °C. For TDP-43_SAA, 2 μg of the pellet was collected using an inoculating loop and transferred to a tube containing 25 μL of PBS. This sample was then further diluted 1:10 in PBS before TDP-43_SAA analysis. For αSyn_SAA, 2 μg of the pellet was resuspended in 17 μL of PBS, and 2 μL of this mixture was further diluted in 38 μL of PBS with 1% Triton X-100 and used for analysis.

**OM SAA analyses**

αSyn_SAA analysis was performed using 384-well optical flat-bottom plates (Thermo Scientifc) and each sample was tested in quadruplicate. Each well was loaded with 49 μL of αSyn_SAA reaction mixture containingrec-αSyn (purchased from rPeptide, S-1001–2) [0.1 mg/mL], HEPES (pH 8.0) [40 mM], sodium citrate [170 mM], and ThT [20 μM]. Two low-binding silica beads (0.8 mm, OPS Diagnostics) and 1μL of each OM sample were added to the well. The plate was then covered with sealing film (Thermo Scientifc), placed into a FLUOstar OMEGA microplate reader (BMG Labtech) and subjected to alternating cycles of shaking (1 min, 600 rpm, single orbital) and incubation (1 min, 42 °C). ThT fluorescence intensities, expressed as AUs, were measured every 30 min at wavelengths of 450 ±10 nm (excitation) and 480 ±10 nm (emission), with a bottom read.

TDP-43_SAA analysis of the OM samples was performed in triplicate following a recent established protocol (Fontana et al.2024) with some modifications. The assay employed a truncated recombinant human TDP-43 protein produced and purified as described in our recent publication (DOI: 10.1186/s13024-025-00833-0). In particular, the analysis was performed using a 96-well optical flat-bottom plate (Thermo Scientific). Each well was loaded with 96 μL of a reaction mixture containing: truncated recombinant TDP-43 protein [0, 05 mg/ml], Tris-HCl pH8 [40mM], Guanidine-HCl pH8 [500mM], SDS [0,002%], water and thioflavin T (ThT) [10uM]. To each well, a 3-mm glass bead (Sigma) and 4 μL of OM samples were added. The plate was sealed with optical sealing film (Thermo Scientific) and placed in a FLUOstar CLARIOstar microplate reader (BMG Labtech). The analysis was run at 42 °C with alternating cycles of shaking (15 s at 100 rpm, double orbital) and incubation (30 min). ThT fluorescence was monitored from the bottom of the plate every 30 minutes using excitation/emission wavelengths of 450 ± 10 nm and 480 ± 10 nm, respectively. Results were reported in arbitrary fluorescence units (AUs). Four healthy OM controls for both SAA assays, two PD cases for αSyn-SAA, and two spinal-onset ALS cases for TDP43-SAA were used to evaluate assay kinetics. For αSyn-SAA, samples were considered positive when fluorescence exceeded 30.000 AU within 20 hours. For TDP43-SAA, samples were considered positive when fluorescence exceeded 50.000 AU within 36 hours (in line with our recent study DOI: 10.1186/s13024-025-00833-0).

**Skin analysis**

Double immunofluorescence was performed on free-floating section of 50 micron. After blocking solution, we incubated overnight the sections with different primary antibodies mixes: 1) anti-p-syn (1:500; rabbit monoclonal, Clone EP1536Y, Abcam) or p-S409/410 TDP-43 (1:1000, rabbit polyclonal, CAC-TIP-PTD-P07, Cosmo Bio USA) and anti-PGP 9.5 (1: 500; mouse monoclonal, Biorad) or anti-vimentin (1:600 mouse monoclonal, Clone V9 M0725, Agilent) or anti-CD68 (1:150 mouse monoclonal, Clone KP1 M0814, Agilent). Then, the sections were incubated in a mix of secondary antibodies: goat anti-mouse conjugated with Alexa 488 and goat anti-rabbit conjugated with Alexa 555 (1:600; 2 hours at room temperature). Finally, DAPI incubation (1:5000, Life technologies) was performed for 10 minutes. The images were acquired using a Zeiss LSM980 confocal microscope. Positive controls were performed on human brain sections (Supplementary Fig. 1a) for p-TDP43 and negative control in healthy subjects for p-TDP43 (supplementary Fig. 1b, c) and p-syn (Supplementary Fig. 1d, e).

**Supplementary Table 1**

| **COGNITIVE DOMAINS** | **TEST** | **T0** | **T1** | **CUT-OFF** |
| --- | --- | --- | --- | --- |
| **GLOBAL COGNITION** | MoCA  CDT | 21,17  4** | 26,64  7,5 | 18,58  5 |
| **LANGUAGE**  Phonemic-based word retrieval  Semantic-based word retrieval | Letter fluency task  Category fluency task | 26  - | 35  51 | 17  25 |
| **MEMORY**  Working memory  Verbal episodic memory  Visuo-spatial memory | Digit span forward  Digit span backward  RAVLT  -immediate recall  -delayed recall  -recognition  -false positive  Babcock Short Story  Immediate recall  Delayed recall  Corsi span  Rey-Osterrieth Complex Figure recall | -  -  -  -  -  -  6  5,4  -  22,7 | 6,23  4,28  44  11,3  14/15  0  6,8  5,9  6,25  21 | <4,26  <2,65  28,53  4,69  3,1  2,39  <3,46  <9,47 |
| **ATTENTION AND EXECUTIVE FUNCTIONS**  Executive abilities  Set-shifting/Attention | FAB  TMT B  TMT B-A  TMT A  MFTC  Execution time  Correct items  False alarms  Accuracy | 12,32**  -  -  -  37,52  13/13  0  1 | 14,4  121  20  101**  69,77  12/13  0  0,96 | 13,5  282  187  93  135,73  2,77  0,869 |
| **VISUOSPATIAL ABILITIES**  Visuo-spatial constructional ability | Rey-Osterrieth Complex Figure copy | 36 | 33,5 | <28,88 |
| **EMOTION AND SOCIAL COGNITION** | SET  SET-IA SET-CI  SET-EA | -  -  -  - | 9,27*  3,18*  3,26*  3,18* | <8,29  <2,34  <2,41  <2,2 |
| **NEUROPSYCHIATRICS SYMPTOMS** | FBI  FBI A  FBI B | -  -  - | 9/72  6/36  3/36 |  |

**Supplementary Table 1. Neuropsychological assessment at baseline (2 years from onset)**

**and follow-up (3 years from onset)**

(adjusted scores listed). * Borderline, ** under cut-off.

p-TDP 43/PGP 9.5/nuclei

p-TDP 43/lipofuscin

p-syn/PGP 9.5/nuclei

d

e

a

b

c

**Supplementary figure 1. p-TDP43 positive and p-TDP43/p-syn negative control.** a: p-TDP-43 aggregates in human brain neurons; b,c: p-TDP-43 is almost negative both in the dermis (b) and in PGP 9.5-positive nerve fibers (c, green) of a healthy control skin biopsy; d, e: p-syn staining is negative within PGP9.5-positive fibers of both a blood vessel (d) and a nerve bundle (e) in the dermis of a healthy control skin biopsy. Scale bar a) 20 μm; b-e) 10 μm.
